# Report of Case of Entozoa in the Kidney

**Published:** 1890-03

**Authors:** W. J. Greene

**Affiliations:** Fort Valley, Ga.


### REPORT OF CASE OF ENTOZOA IN THE KIDNEY

---

BY W. J. GREENE, M. D., FORT VALLEY, GA.

---

On the 13th inst. I was called to see Mr. W. H. Weston, (Union Telegraph operator in our town).

When I arrived he had been taken from the telegraph office to his home. I found him suffering intense pain in the left side, running in a line from the left kidney towards the bladder. My diagnosis at first was passage of "Renal Calculus." He told me that when first taken in the office his pain in the region above designated was sudden and intense. His bladder became suddenly filled (as he thought with urine); upon emptying the bladder he discharged about one pint of blood, and then discovered hanging from the urethra something like a worm, which he pulled out and left upon the floor of his office (which I was never able to find). His suffering continued severe a good portion of the night. I kept him under the influence of anodynes, chloroform, hot poultices, hot cloths, etc., until two or three o'clock

A. M., at which time he said he had a painful desire to void urine. Upon examination I found in the urine voided a worm about three and one-half inches long, wriggling about in his native element. Prof. Robley Dungleson says the presence of entozoa in the urinary organs of man is not common, but they are very often met with in the inferior animals.

Prof. Gross says they are exceedingly common in the hog. With an experience of thirty-eight years in the practice of medicine this is the first case of the kind that has ever come under my observation. Since the passage of the worm Mr. Weston has suffered little or no inconvenience, and is up and attending his usual vocation.

---

MRÆCK. "Treatment of erysipelas."—*Gaz. Hebd. de Med.*, November 15, 1889.

The treatment of erysipelas by the external application of germicides is growing in favor. Carbolic acid, though used to some extent, has the disadvantage of being in itself an irritant if in sufficient strength to modify the disease. Creolin, which is undoubtedly a powerful germicide, is recommended for the purpose, either alone or in combination with iodoform, in the former case in the proportion of 3 parts to 25 of lanolin, in the latter of 1 part creolin, 4 of iodoform, and 10 of lanolin. It is applied not only on the erysipelatous area, but for an inch or more beyond its boundaries. To prevent rubbing off and to assist absorption, the whole is covered with gutta-percha. The iodine which is set free in the combination is supposed to be an important agent, as well as the creolin. The treatment is continued for two or three days after the disappearance of the redness, and the results are reported to be very good.—*Medical Chronicle.*

